# Mannose-binding lectin does not explain the course and outcome of pregnancy in rheumatoid arthritis

**DOI:** 10.1186/ar3231

**Published:** 2011-01-31

**Authors:** Fleur E van de Geijn, Yaël A de Man, Manfred Wuhrer, Sten P Willemsen, André M Deelder, Johanna MW Hazes, Radboud JEM Dolhain

**Affiliations:** 1 Department of Rheumatology, Erasmus University Medical Center Rotterdam, Dr. Molewaterplein 50, NL-3015 GE, Rotterdam, The Netherlands; 2 Biomolecular Mass Spectrometry Unit, Department of Parasitology, Leiden University Medical Center, Albinusdreef 2, NL-2333 ZA, Leiden, The Netherlands; 3 Department of Biostatistics, Erasmus University Medical Center Rotterdam, Dr. Molewaterplein 50, NL-3015 GE, Rotterdam, The Netherlands

## Abstract

**Introduction:**

Rheumatoid arthritis (RA) improves during pregnancy and flares after delivery. It has been hypothesized that high levels of the complement factor mannose-binding lectin (MBL) are associated with a favourable disease course of RA by facilitating the clearance of pathogenic immunoglobulin G (IgG) lacking galactose sugar moieties. During pregnancy, increased galactosylation of IgG and simultaneously increased MBL levels can be observed, with the latter being strictly related to maternal MBL genotypes. Therefore, increased MBL levels in concert with increased IgG galactosylation may be associated with pregnancy-induced improvement of RA. The objective of this study was to investigate whether MBL genotypes are associated with changes in RA disease activity and with changes in IgG galactosylation during pregnancy and in the postpartum period. We also studied the association between MBL genotypes and pregnancy outcomes in RA.

**Methods:**

Serum from 216 patients with RA and 31 healthy controls participating in the Pregnancy-induced Amelioration of Rheumatoid Arthritis (PARA) Study was collected before, during and after pregnancy. IgG galactosylation was determined by performing matrix-assisted laser desorption/ionization time of flight mass spectrometry. Disease activity was determined using the internationally recognized Disease Activity Score 28 (DAS28). MBL genotypes were determined. The pregnancy outcome measures studied were gestational age, birth weight, miscarriage and hypertensive disorders.

**Results:**

No association was found between the MBL genotype groups and changes in RA disease activity (*P *= 0.89) or changes in IgG galactosylation (patients, *P *= 0.75, and controls, *P *= 0.54) during pregnancy and in the postpartum period. Furthermore, MBL genotype groups were not related to the studied pregnancy outcome measures.

**Conclusions:**

This study does not provide evidence for a role for MBL in the improvement of RA during pregnancy or for a role for MBL in pregnancy outcome.

## Introduction

Pregnancy is the only natural situation that results in spontaneous improvement of rheumatoid arthritis (RA) and a flare of the disease after delivery in a substantial number of patients. Insight into the mechanism of this phenomenon may therefore not only enlarge our knowledge of the phenomenon of pregnancy-induced remission in RA but also may contribute to a better understanding of the pathogenic mechanisms underlying RA in general. It has been hypothesized that high levels of the complement factor mannose-binding lectin (MBL) are associated with a favorable disease course of RA by binding to and hence facilitating the clearance of pathogenic immunoglobulin G (IgG), which lacks galactose sugar moieties (agalactosyl IgG) [1].

MBL is the initiator of the innate immunity lectin complement pathway, and its serum levels are highly variable between individuals because of the presence of single-nucleotide polymorphisms (SNPs) in the promoter region and in exon 1 of the *MBL2 *gene. It has been shown that MBL levels are markedly increased during pregnancy and that this increase is strictly related to the high maternal MBL production genotypes [2].

It has been shown *in vitro *that MBL can bind to pathogenic agalactosyl IgG [3]. In patients with RA, levels of agalactosyl IgG decline during pregnancy, and hence galactosylation increases simultaneously with improvement of RA disease activity. Postpartum galactosylation of IgG decreases, which is associated with the well-known flare of RA disease activity after delivery [4,5]. These changes in galactosylation have been shown both for IgG1 and for IgG2 [4]. Therefore, it has been suggested that during pregnancy the MBL protein could play a role in the clearance of pathogenic (agalactosyl IgG) immune complexes by serving as a scavenger molecule with an anti-inflammatory role [1]. This would decrease RA disease activity during pregnancy, and consequently low levels of MBL could be responsible for the postpartum RA flare [2]. It should be noted, though, that according to the literature, MBL might play a dual role in the pathogenesis of RA. Namely, a proinflammatory role of MBL has been described whereby its binding to agalactosyl IgG can also activate the complement system [3] and therefore lead to increased inflammation [1]. However, we hypothesize that the anti-inflammatory role of MBL might be more prevalent during pregnancy in patients with RA.

Apart from the role of MBL in RA, MBL has also been associated with pregnancy outcomes such as gestational age, birth weight, recurrent miscarriages, risk for chorioamnionitis and severe (or recurrent) preeclampsia in healthy individuals [6-9]. Whether the same holds true for RA is unknown.

We therefore aim to provide evidence for a role for MBL not only in the improvement of RA during pregnancy but also in the pathogenesis of RA in general by investigating whether high MBL production genotypes are associated with improvement of RA disease activity, and associated with changes in IgG galactosylation during pregnancy and the postpartum flare. Moreover, the possible association between MBL genotypes and pregnancy outcomes in RA is studied. We have measured MBL genotypes because they have a very good correlation with MBL serum levels in healthy individuals [10] and patients with RA [11] as well as during pregnancy [2].

## Materials and methods

### Study population

The current study is embedded within the Pregnancy-induced Amelioration of Rheumatoid Arthritis (PARA) Study, which is a prospective cohort study on pregnancy and RA [12]. In this study, patients with RA are visited preferably before pregnancy, three times during pregnancy and three times postpartum. The disease activity of RA was scored using the internationally recognized Disease Activity Score 28 (DAS28) with three variables (swollen joint count, tender joint count and C-reactive protein (CRP) level), since this variant of the DAS28 is the most reliable during pregnancy [13]. Some patients were analyzed during more than one pregnancy. Controls were followed from the first trimester of pregnancy onwards. Data from a total of 216 Caucasian patients with RA (patients) and 31 healthy pregnant Caucasian volunteers without an adverse obstetric history (controls) were included in the study. Informed patient consent was obtained for the study. The study is in compliance with the Helsinki Declaration and was approved by the Ethics Review Board at the Erasmus MC University Medical Center, Rotterdam, the Netherlands.

### Data collection

Available data for patients differed for each research question. To investigate whether MBL genotypes are associated with changes in RA disease activity, data for a maximum of 181 patients were available. Patients who experienced a miscarriage were excluded. To investigate whether MBL genotypes are associated with changes in IgG galactosylation, data for a maximum of 145 patients were available. The association between MBL genotypes and pregnancy outcomes, including miscarriages, could be studied in 214 patients. For the analyses of the pregnancy outcome measures: birth weight and gestational age, non-Caucasians, twin pregnancies and pregnancies that resulted in the birth of a child with a malformation were excluded, resulting in 184 patients to be studied. Data were analyzed with or without the patients who participated twice or more.

### Categorization of disease activity and clinical response

In accordance with the European League Against Rheumatism (EULAR) criteria, remission of RA was defined as DAS28 <2.6 and intermediate and high disease activity as DAS28 >3.2. Improvement of disease activity during pregnancy was defined according to the EULAR criteria as 'good', 'moderate' (combined with 'responders') or 'nonresponders'. In line with the EULAR criteria, the response criteria can be applied only to those patients with an initial DAS28 >3.2 in the first trimester (*n *= 84). Deterioration of disease activity after delivery was defined according to so-called reversed EULAR criteria [12]. Since there is no baseline DAS28 requirement for these criteria, this classification was applied to all patients. An early flare was defined as the beginning of deterioration between 6 weeks and 3 months postpartum, and a late flare was defined as disease deterioration between 3 and 6 months postpartum.

### MBL genotyping

Genotyping was performed using LightCycler Real-Time PCR (Roche Applied Science, Almere, The Netherlands) techniques as described previously [2]. Genotyping included the wild type (A allele) and the three SNPs of the first exon of the structural gene: codon 52 (D allele, rs5030737), codon 54 (B allele, rs1800450) and codon 57 (C allele, rs1800451) and two of the SNPs in the promoter region, codon -550 (H/L, rs11003125) and codon -221 (X/Y, rs7096206) of the *MBL2 *gene. On the basis of the haplotypes, individuals can be categorized into three groups that correlate best with MBL serum levels [10,11]: the high MBL production group, A; the intermediate MBL production group, B; and the low or deficient MBL production group, C. Using such an approach in patients with RA, a very good correlation between MBL genotype groups and MBL serum concentrations has been shown (Spearman's ρ = 0.82, *P *< 0.0001) [11].

### IgG galactosylation analysis

IgG was purified from the sera of patients and controls as described previously [4,14]. Next, IgG galactosylation was analysed by performing matrix-assisted laser desorption/ionization time of flight mass spectrometry to detect tryptic glycopeptides, and mass spectra of IgG1 and IgG2 were processed using FlexAnalysis software (Bruker Daltonics, Wormer, The Netherlands).

### Pregnancy outcome definitions

Preterm birth was defined as gestational age <37 weeks of gestation, and low birth weight was defined as a birth weight <2,500 g. As described before, the birth weights analyzed were corrected for gestational age and the sex of the child by using the birth weight standard deviation (SD) score. Birth weight SD scores as well as uncorrected birth weights are added to the analyses [15]. Hypertensive disorders were scored according to the criteria of the International Society for the Study of Hypertension and Pregnancy [7]. Miscarriage was scored in case of a spontaneous loss of a pregnancy before the 20th week.

### Statistical analysis

Statistical analysis was performed using SPSS version 15.0 software (SPSS, Inc., Chicago, IL, USA) and SAS version 9.1 software (SAS Inc., Cary, NC, USA). A two-sided *P *value ≤0.05 was considered statistically significant. The disease activity (DAS28, patients) and galactosylation profile (patients and controls) were estimated using a linear mixed model. By using this model, we investigated possible associations between the MBL genotype groups and DAS28 or IgG galactosylation. χ^2 ^analysis was performed to compare MBL genotype groups of responders versus nonresponders, patients with a flare versus no flare postpartum, patients which had a miscarriage versus those who had not had a miscarriage, patients which had a preterm birth versus those who did not have a preterm birth and patients with a child with low birth weight versus those who did not have a child with low birth weight.

Logistic regression analysis was performed for dichotomous variables, and linear regression analysis was performed for linear data. On the basis of the literature, we considered the following variables *a priori *to be possible confounders (when applicable): gestational age, maternal smoking during pregnancy, maternal age at delivery, sex of the child, prednisone use during the first trimester, parity and disease activity in the first trimester (DAS28). First, simple regression analyses were performed to determine which confounders had to be included in the multiple regression analyses.

## Results

### Clinical characteristics of the study group

The characteristics of the patients and controls are given in Table 1.

**Table 1:** Cohort characteristics^a^

Cohort	Patients (*n *= 214)	Controls (*n *= 31)
MBL genotype group A, *n *(%)	114 (53.3)	16 (51.6)
MBL genotype group B, *n *(%)	59 (27.6)	8 (25.8)
MBL genotype group C, *n *(%)	41 (19.2)	7 (22.6)
Number of Caucasians, *n *(%)	207 (96.7)	31 (100)
Number of nulliparous women, *n *(%)	113/214 (52.8)	14/31 (45.2)
Mean age at delivery, yr (± SD) (range)	32.5 ± 3.7 (21.9 to 40.6)	32.1 ± 4.5 (24.2 to 40.1)
Mean gestational age at delivery, wk (range)	39.4 (31.4 to 42.1)	40.0 (34.7 to 42.0)
Smoking during pregnancy, *n *(%)	6/206 (2.9)	3/31 (9.7)
Miscarriage, *n *(%)	23 (10.7)	-
Hypertension, *n *(%)	25/210 (11.7%)	2 (6.5)
Preeclampsia, *n *(%)	4/210 (1.9)	1 (3.2)
Anti-CCP-positive, *n *(%)	134/213 (62.9)	-
Rheumatoid factor (IgM)-positive, *n *(%)	161/214 (75.1)	-
Erosive disease, *n *(%)	136/210 (64.8)	
Median disease duration at delivery, yr (range)	7.9 (0.7 to 29.0)	-
Use of prednisone in first trimester, *n *(%)	60/164 (36.6%)	
Median number of DMARDs (including prednisone) prior to conceive (min-max)	2.3 (0-6)	-
Use of methotrexate prior to conception, *n *(%)	120/212 (56.6)	-
DAS28-CRP3 >3.2 in first trimester, *n *(%)	84/155 (54.2)	-
Classification of disease activity during pregnancy		
Good response or moderate response, *n *(%)	40/84 (47.6)	-
No response, *n *(%)	44/84 (52.4)	-
Classification of disease activity during postpartum period (early flare)		
Severe or moderate deterioration, *n *(%)	39/167^b ^(23.4)	-
No deterioration, *n *(%)	128/167^b ^(76.6)	-
Classification of disease activity during postpartum period (late flare)		
Severe or moderate deterioration, *n *(%)	28/152^b ^(10.3)	-
No deterioration, *n *(%)	124/152^b ^(45.6)	-

^a^MBL, mannose-binding lectin; n, number; SD, standard deviation; anti-CCP, anti-cyclic citrullinated peptide; IgM, immunoglobulin M; DMARDs, disease-modifying antirheumatic drugs; DAS28-CRP3, Disease Activity Score 28 using three variables, including C-reactive protein; ^b^cases are missing because DAS score data are missing in a proportion of patients.

### Accuracy genotyping procedure

MBL genotypes were determined in 216 patients and 31 controls. In two patients, the promoter SNPs could not be determined. Therefore, these patients could not be assigned to one of the MBL genotype groups and were excluded from analyses, resulting in a total of 214 patients and 31 controls to be analyzed.

### No association of MBL genotype groups and RA disease activity

**Figure 1:**
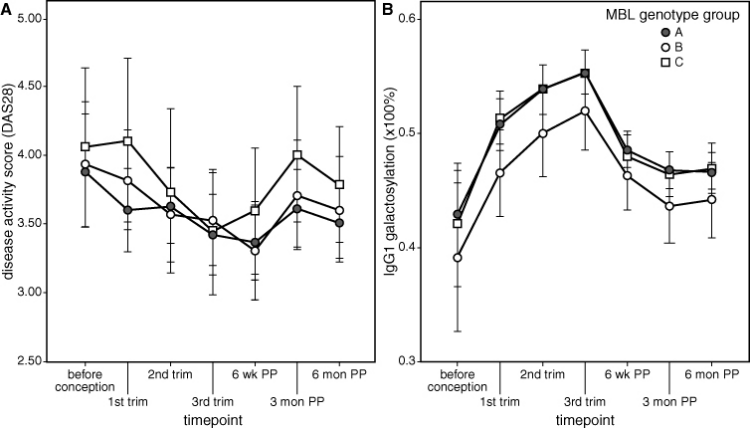
**Disease activity score and immunoglobulin G galactosylation in relation to mannose-binding lectin genotype groups**. **(a) **Mean rheumatoid arthritis (RA) disease activity score 28 (DAS28) during pregnancy and postpartum (PP) in relation to mannose-binding lectin (MBL) production genotype groups A (high), B (intermediate) and C (low). No significant difference in DAS28 levels is observed between MBL genotype groups A, B and C at all time points during pregnancy and postpartum (*P *= 0.899). **(b) **Mean Immunoglobulin G1 (IgG1) galactosylation (×100%) of patients with RA during pregnancy and postpartum per MBL production genotype group. No significant difference in IgG galactosylation levels is observed between MBL genotype groups A, B and C at all time points during pregnancy and postpartum (*P *= 0.75). Data for IgG2 galactosylation and for the controls show similar results (data not shown). The vertical bars indicate the 95% confidence intervals. trim, trimester of pregnancy; wk, weeks; PP, postpartum; mon, months.

No significant differences in DAS28 levels were observed between MBL genotype groups A, B and C at all time points during pregnancy and the postpartum period (*P *= 0.899) (Figure 1a). Also, no differences were observed between the MBL genotype groups when patients were categorized into responder and nonresponder groups during pregnancy, among patients who had an early or late postpartum flare and among patients who did not have a postpartum flare (responders vs. nonresponders: MBL genotype group A versus MBL groups B and C, odds ratio (OR) 0.91, 95% confidence interval (95% CI) 0.58 to 1.42; early flare vs. no flare: MBL genotype group A versus MBL genotypes B and C, OR 0.69, 95% CI 0.39 to 1.25; late flare vs. no flare: MBL genotype group A versus MBL genotype groups B and C, OR 1.00, 95% CI 0.51 to 1.98). Similar results were found when groups A, B and C were analyzed separately (data not shown) and when patients who participated twice or more were excluded.

### No association of MBL genotype groups and IgG galactosylation changes

No significant differences in IgG1 galactosylation levels were observed between MBL genotype groups A, B and C at all time points during the pregnancy and postpartum periods as shown in Figure 1b (*P *= 0.75, patients). Similar nonsignificant results were obtained for IgG2 as well as in controls (data not shown). Univariate and multivariate analyses revealed that MBL genotype groups do not affect IgG galactosylation levels, even when corrections for possible confounders such as medication use, disease activity and clinical characteristics are applied.

### No association of MBL genotype groups and pregnancy outcome in RA

In RA, the gestational age or birth weight did not differ significantly among the MBL genotype groups (*P *= 0.78 and *P *= 0.95, respectively). Accordingly, there was a similar distribution of preterm birth and low birth weight infants among MBL genotype groups (*P *= 0.75 and *P *= 0.68, respectively). The distribution of miscarriages (23 of 201 patients) was also not significantly different among the MBL genotype groups (*P *= 0.81).

All logistic regression and linear regression analyses could not show an association between MBL genotype groups and the pregnancy outcome measures preterm birth, low birth weight, hypertensive disorders, miscarriage and gestational age, birth weight SD score or birth weight (Tables 2 and 3), even after correction for multiple possible confounders as described above. Subgroup analysis for nulliparous women as well as for patients who did not use prednisone in the first trimester of pregnancy did not reveal any effect of MBL on gestational age, birth weight or birth weight SD score (data not shown). Grouping of the intermediate and low MBL genotype groups B and C in all linear and logistic analyses did not reveal a different effect (data not shown).

**Table 2:** Regression analysis of mannose-binding lectin genotype groups and pregnancy outcome measures based on continuous variables^a^

	MBL genotype groups A, B and C (three strata)			MBL genotype group A vs. B plus C (dichotomous)		
		
Variable stratified	β-coefficient	*P *value	*n*	β-coefficient	*P *value	*n*
Gestational age, wk						
No correction	-0.062	0.739	156	-0.085	0.767	156
Correction for all confounders	-0.27	0.199	126	-0.363	0.260	126
Birth weight, g						
No correction	-25.69	0.672	157	-6.16	0.947	157
Correction for all confounders	-81.76	0.205	127	-69.56	0.480	127
Birth weight SD score						
No correction	-0.015	0.896	156	0.03	0.865	156
Correction for all confounders	-0.052	0.671	126	0.004	0.983	126

^a^Continuous variable outcome measures include gestational age, birth weight, birth weight SD score in patients with rheumatoid arthritis. MBL, mannose-binding lectin; A, B and C, MBL production genotype groups A (high), B (intermediate) and C (low); SD, standard deviation.

**Table 3:** Regression analysis of mannose-binding lectin genotype groups and pregnancy outcome measures based on dichotomous variables^a^

	MBL genotype groups A, B and C (three strata)			MBL genotype group A vs. B plus C (dichotomous)		
		
Variable stratified	OR	95% CI	*n*	OR	95% CI	*n*
Miscarriage						
No correction	1.13	0.64 to 1.99	21/205	1.31	0.53 to 3.23	21/205
Correction for all confounders	0.99	0.53 to 1.83	20/178	1.12	0.43 to 2.87	20/178
Hypertension						
No correction	0.93	0,53 to 1.66	23/174	0.72	0.30 to 1.78	23/174
Correction for all confounders	0.76	0.36 to 1.61	16/127	0.58	0.18 to 1.83	16/127
Gestational age (<37 wk)						
No correction	1.30	0.68 to 2.58	14/157	1.20	0.40 to 3.60	14/157
Correction for all confounders	2.12	0.80 to 5.60	13/127	2.38	0.47 to 12.1	13/127
Low birth weight (<2,500 g)						
No correction	0.93	0.40 to 2.28	10/158	0.76	0.21 to 2.82	10/158
Correction for all confounders	0.92	0.30 to 2.90	9/127	0.87	0.14 to 5.38	9/127

^a^Dichotomous variable outcome measures include miscarriage, hypertension, gestational age and low birth weight in patients with rheumatoid arthritis. MBL, mannose-binding lectin; A, B and C, MBL production genotype groups A (high), B (intermediate) and C (low); OR, odds ratio; 95% CI, 95% confidence interval.

## Discussion

In this study, no association was found between MBL genotype groups and improvement of RA during pregnancy or with levels of IgG galactosylation and changes thereof, thereby raising questions about a role for MBL not only in the pregnancy-induced improvement of RA in particular but also for a more general role of MBL in the pathogenesis of RA. Moreover, MBL genotype groups did not show statistically significant associations with gestational age, birth weight, miscarriage and hypertensive disorders in the pregnancies of women with RA.

Previously, high MBL levels were associated with less severe disease in RA [1], indicating an anti-inflammatory role for MBL in RA. It has been suggested that the beneficial effect of MBL results from its binding to the pathogenic agalactosyl IgG antibodies and that MBL might therefore function as a scavenger molecule involved in the efficient removal of pathogenic agalactosyl IgG-containing immune complexes [1]. It should be noted, though, that according to the literature, MBL might play a dual role in the pathogenesis of RA. Namely, a proinflammatory role of MBL also has been described on the basis of its binding to agalactosyl IgG, which can activate the complement system [3] and therefore lead to increased inflammation [1]. However, we hypothesize that the anti-inflammatory role of MBL is more prevalent during pregnancy in patients with RA. Because pharmaceutical induction of MBL is not yet possible, this hypothesis cannot be properly tested *in vivo*. However, during pregnancy, MBL levels increase and RA disease activity improves along with a decrease in the levels of pathogenic agalactosyl IgG. All of these factors make pregnancy in RA the ideal 'experiment of nature' to gain support for the aforementioned hypothesis. Nevertheless, even this ideal setting did not support a role for MBL in the pathogenesis of RA. These results are in line with a recent cross-sectional study that demonstrated no association between MBL genotypes and disease susceptibility and severity in RA [11].

Alternative hypotheses have been proposed to explain the pregnancy-induced improvement of RA, such as the induction of regulatory T cells, immunomodulatory properties of pregnancy hormones, a shift towards a Th2-associated cytokine profile and immunosuppression as a result of increased fetal-maternal human leukocyte antigen disparity [16]. It is likely that multiple mechanisms may work in concert to induce RA improvement during pregnancy.

Finally, the possible association between MBL genotypes and pregnancy outcomes was investigated. Previously published literature demonstrated in healthy individuals that MBL is associated with pregnancy outcomes such as preterm birth, low birth weight, recurrent miscarriages, risk for chorioamnionitis and more severe or recurrent preeclampsia [6-9]. Our study in patients with RA showed no significant association between MBL genotype groups and the pregnancy outcome measures gestational age, birth weight, miscarriage and hypertensive disorders. In line with a previous study on the effect of MBL on gestational age in healthy women [6], an association was found between preterm birth and the maternal high MBL production genotype group A, although in the present study of patients with RA, it did not reach statistical significance (OR, 2.38; 95% CI, 0.47 to 12.1). With regard to the other pregnancy outcome measures, such as preeclampsia, the present study obviously lacks power.

## Conclusions

This study does not suggest a role for MBL in the phenomenon of pregnancy-induced improvement of RA or in the pathogenesis of RA in general. Future studies should focus on other mechanisms to explain the pregnancy-induced remission of RA and the postpartum flare.

## Abbreviations

CCP: cyclic citrullinated peptide; CI: confidence interval; CRP: C-reactive protein; DAS28: Disease Activity Score 28; DMARD: disease-modifying antirheumatic drug; EULAR: European League Against Rheumatism; Gal: galactose; IgG: immunoglobulin G; MALDI-TOF-MS: matrix-assisted laser desorption/ionization time of flight mass spectrometry; MBL: mannose-binding lectin; mon, months; OR: odds ratio; PARA Study: Pregnancy-induced Amelioration of Rheumatoid Arthritis Study; PCR: polymerase chain reaction; PP: postpartum; RA: rheumatoid arthritis; SAS: statistical analysis software; SD: standard deviation; SNP: single-nucleotide polymorphism; SPSS: Statistical Package for the Social Sciences; trim: trimester of pregnancy; wk: weeks.

## Competing interests

The authors declare that they have no competing interests

## Authors' contributions

FG and RD had full access to all of the data in the study and take responsibility for the integrity of the data and the accuracy of the data analysis. FG, MW, YM, AD, MH and RD designed the study. FG, MW and YM were involved in the acquisition of the data. FG, MW, SW, MH and RD analyzed the matrix-assisted laser desorption/ionization time of flight mass spectrometry data and interpreted the data. The manuscript was prepared by FG, MW, SW, YM, AD, MH and RD. FG and SW did the statistical analyses. All authors read and approved the final manuscript.

## References

[B1] Garred P Madsen HO Marquart H Hansen TM Sorensen SF Petersen J Volck B Svejgaard A Graudal NA Rudd PM Dwek RA Sim RB Andersen V Two edged role of mannose binding lectin in rheumatoid arthritis: a cross sectional study J Rheumatol 2000 27 26 34 10648014

[B2] Van de Geijn FE Roos A De Man YA Laman JD De Groot CJ Daha MR Hazes JM Dolhain RJ Mannose-binding lectin levels during pregnancy: a longitudinal study Hum Reprod 2007 22 362 371 10.1093/humrep/del392 17099209

[B3] Malhotra R Wormald MR Rudd PM Fischer PB Dwek RA Sim RB Glycosylation changes of IgG associated with rheumatoid arthritis can activate complement via the mannose-binding protein Nat Med 1995 1 237 243 10.1038/nm0395-237 7585040

[B4] Van de Geijn FE Wuhrer M Selman MH Willemsen SP de Man YA Deelder AM Hazes JM Dolhain RJ Immunoglobulin G galactosylation and sialylation are associated with pregnancy-induced improvement of rheumatoid arthritis and the postpartum flare: results from a large prospective cohort study Arthritis Res Ther 2009 11 R193 10.1186/ar2892 20015375 PMC3003510

[B5] Alavi A Arden N Spector TD Axford JS Immunoglobulin G glycosylation and clinical outcome in rheumatoid arthritis during pregnancy J Rheumatol 2000 27 1379 1385 10852257

[B6] Van de Geijn FE Dolhain RJ van Rijs W Willemsen SP Hazes JM de Groot CJ Mannose-binding lectin genotypes are associated with shorter gestational age: an evolutionary advantage of low MBL production genotypes? Mol Immunol 2008 45 1514 1518 10.1016/j.molimm.2007.08.021 17942155

[B7] Van de Geijn FE Dolhain RJ van Rijs W Hazes JM de Groot CJ Mannose-binding lectin genotypes and pre-eclampsia: a case-control study Hum Immunol 2007 68 888 893 10.1016/j.humimm.2007.10.002 18082567

[B8] Christiansen OB Nielsen HS Lund M Steffensen R Varming K Mannose-binding lectin-2 genotypes and recurrent late pregnancy losses Hum Reprod 2009 24 291 299 10.1093/humrep/den377 18927129

[B9] Than NG Romero R Erez O Kusanovi JP Tarca AL Edwin SS Kim JS Hassan SS Espinoza J Mittal P Mazaki-Tovi S Friel L Gotsch F Vaisbuch E Camacho N Papp Z A role for mannose-binding lectin, a component of the innate immune system in pre-eclampsia Am J Reprod Immunol 2008 60 333 345 10.1111/j.1600-0897.2008.00631.x 18727690 PMC2775464

[B10] Madsen HO Garred P Thiel S Kurtzhals JA Lamm LU Ryder LP Svejgaard A Interplay between promoter and structural gene variants control basal serum level of mannan-binding protein J Immunol 1995 155 3013 3020 7673719

[B11] Van de Geijn FE Hazes JM Geleijns K Emonts M Jacobs BC Dufour-van den Goorbergh BC Dolhain RJ Mannose-binding lectin polymorphisms are not associated with rheumatoid arthritis: confirmation in two large cohorts Rheumatology 2008 47 1168 1171 10.1093/rheumatology/ken226 18562462

[B12] De Man YA Dolhain RJ van de Geijn FE Willemsen SP Hazes JM Disease activity of rheumatoid arthritis during pregnancy: results from a nationwide prospective study Arthritis Rheum 2008 59 1241 1248 10.1002/art.24003 18759316

[B13] De Man YA Hazes JM van de Geijn FE Krommenhoek C Dolhain RJ Measuring disease activity and functionality during pregnancy in patients with rheumatoid arthritis Arthritis Rheum 2007 57 716 722 10.1002/art.22773 17530669

[B14] Wuhrer M Stam JC van de Geijn FE Koeleman CA Verrips CT Dolhain RJ Hokke CH Deelder AM Glycosylation profiling of immunoglobulin G (IgG) subclasses from human serum Proteomics 2007 7 4070 4081 10.1002/pmic.200700289 17994628

[B15] De Man YA Hazes JM van der Heide H Willemsen SP de Groot CJ Steegers EA Dolhain RJ Association of higher rheumatoid arthritis disease activity during pregnancy with lower birth weight: results of a national prospective study Arthritis Rheum 2009 60 3196 3206 10.1002/art.24914 19877045

[B16] Ostensen M Villiger PM The remission of rheumatoid arthritis during pregnancy Semin Immunopathol 2007 29 185 191 10.1007/s00281-007-0072-5 17621703

